# MicroRNA expression profile of human umbilical vein endothelial cells in response to coxsackievirus A10 infection reveals a potential role of miR-143-3p in maintaining the integrity of the blood–brain barrier

**DOI:** 10.3389/fcimb.2023.1217984

**Published:** 2023-07-28

**Authors:** Yajie Hu, Fengxian Cui, Shenglan Wang, Chen Liu, Shengxiong Zhang, Ruiqi Wang, Jie Song, Yunhui Zhang

**Affiliations:** ^1^ Department of Pulmonary and Critical Care Medicine, The First People’s Hospital of Yunnan Province, Kunming, China; ^2^ The Affiliated Hospital of Kunming University of Science and Technology, Kunming, Yunnan, China; ^3^ Yunnan Provincial Key Laboratory of Clinical Virology, The First People’s Hospital of Yunnan Province, Kunming, China; ^4^ Department of Thoracic Surgery, The First People’s Hospital of Yunnan Province, Kunming, China; ^5^ Institute of Medical Biology, Chinese Academy of Medical Science and Peking Union Medical College, Yunnan Key Laboratory of Vaccine Research and Development on Severe Infectious Diseases, Kunming, China

**Keywords:** hand, foot, and mouth disease (HFMD), coxsackievirus A10 (CV-A10), microRNAs (miRNAs), high-throughput sequencing, blood–brain barrier (BBB)

## Abstract

Coxsackievirus A10 (CV-A10) has been one of the main etiologies of hand, foot, and mouth disease (HFMD) epidemics in recent years and can cause mild to severe illness and even death. Most of these severe and fatal cases were closely associated with neurological impairments, but the potential mechanism of neuropathological injury triggered by CV-A10 infection has not been elucidated. MicroRNAs (miRNAs), implicated in the regulation of gene expression in a post-transcriptional manner, play a vital role in the pathogenesis of various central nervous system (CNS) diseases; therefore, they serve as diagnostic biomarkers and are emerging as novel therapeutic targets for CNS injuries. To gain insights into the CV-A10-induced regulation of host miRNA-processing machinery, we employed high-throughput sequencing to identify differentially expressed miRNAs in CV-A10-infected human umbilical vein endothelial cells (HUVECs) and further analyzed the potential functions of these miRNAs during CV-A10 infection. The results showed that CV-A10 infection could induce 189 and 302 significantly differentially expressed miRNAs in HUVECs at 24 and 72 hpi, respectively, compared with the uninfected control. Moreover, the expression of four selected miRNAs and their relevant mRNAs was determined to verify the sequencing data by quantitative reverse transcription–polymerase chain reaction (RT–qPCR) methods. After that, gene target prediction and functional annotation revealed that the targets of these dysregulated miRNAs were mostly enriched in cell proliferation, signal transduction, cAMP signalling pathway, cellular response to interleukin-6, ventral spinal cord interneuron differentiation, negative regulation of glial cell differentiation, neuron migration, positive regulation of neuron projection development, etc., which were primarily involved in the processes of basic physiology, host immunity, and neurological impairments and further reflected vital regulatory roles of miRNA in viral pathogenicity. Finally, the construction of a miRNA-regulated network also suggested that the complex regulatory mechanisms mediated by miRNAs might be involved in viral pathogenesis and virus–host interactions during CV-A10 infection. Furthermore, among these dysregulated miRNAs, miR-143-3p was demonstrated to be involved in the maintenance of blood–brain barrier (BBB) integrity.

## Introduction

Hand, foot, and mouth disease (HFMD) is a contagious disease commonly found in pediatrics, mainly occurring among young children in school and daycare settings (Saguil et al., 2019). Usually, HFMD presents as a self-limited febrile illness with malaise, oral ulcerations causing throat or mouth pain, and a vesicular exanthem found on the hands and feet, but a small proportion of children may develop severe complications, such as herpangina, viral encephalitis, myocarditis, acute flaccid paralysis, and neurorespiratory syndrome (Chang et al., 2018; Esposito and Principi, 2018). Historically, outbreaks of HFMD are primarily caused by enterovirus 71 (EV-A71) and coxsackievirus A16 (CV-A16) (Chang et al., 2018; Esposito and Principi, 2018). However, according to epidemiological data on HFMD in recent years, coxsackievirus A6 (CV-A6) and coxsackievirus A10 (CV-A10) have become the new and major agents in HFMD outbreaks and sporadic cases globally (Kimmis et al., 2018; Bian et al., 2019). Moreover, CV-A10 infection has been demonstrated to usually lead to a high incidence of fatal neurologic or cardiopulmonary complications similar to that of EV-A71 (Bian et al., 2019; Duan et al., 2021). An inactivated EV-A71 vaccine has successfully entered the market and shown high efficacy against EV-A71-associated HFMD (Mao et al., 2016), but this vaccine does not exert effective cross-protection against infections with other enteroviruses, including CV-A10 (Aswathyraj et al., 2016; Fang and Liu, 2018). At present, there is no specific drug for effective pharmacological intervention for patients with CV-A10 infection and no safe and effective vaccine to prevent CV-A10 infection (Aswathyraj et al., 2016). Hence, it is urgent for us to further strengthen the basic theoretical research on the pathogenesis of CV-A10 infection to find key targeted drug molecules for follow-up research and development of CV-A10-associated HFMD-targeted therapeutic drugs.

MicroRNAs (miRNAs) are a large family of non-coding RNA molecules 20–22 nucleotides in length that have emerged in recent years as central regulators of eukaryotic gene expression at the post-transcriptional level (Cai et al., 2009). Studies on the influence of post-transcriptional control on viral infections have demonstrated that miRNAs can affect RNA virus replication and pathogenesis through direct binding to the RNA virus genome or virus-mediated changes in the host transcriptome (Trobaugh and Klimstra, 2017). For example, miR-24 and miR-93 mediate antiviral defense against vesicular stomatitis virus (VSV) infection in mice by targeting the viral large protein (L protein) and phosphoprotein (P protein) genes, respectively (Otsuka et al., 2007). MiR-138 promotes the latency of herpes simplex virus type 1 (HSV-1) by repressing the immediate early proteins of HSV-1, such as ICP0 (Sun et al., 2021). MiR-122, a very abundant liver-specific miRNA, regulates fatty acid and cholesterol biosynthesis and ultimately facilitates the replication of the hepatitis C virus (HCV) (Ono et al., 2020). Therefore, the above studies have indicated that miRNAs are a class of regulatory RNAs in host–pathogen interactions (Cai et al., 2009). There is also evidence that enteroviruses alter host miRNAs to promote the progression of pathogenesis (Engelmann et al., 2018). For instance, coxsackievirus B3 (CV-B3)-induced miR-21 expression directly affects intercalated disk structure, which might be a new mechanism in modulating cell–cell interactions of cardiomyocytes during CV-B3 infection (Ye et al., 2014). EV-A71-infected human oral epithelial cells selectively package high levels of miR-30a into exosomes and functionally transfer miR-30a to receptor macrophages with the help of exosome release, thereby inhibiting the production of type I interferon in receptor cells by targeting MyD88 and further accelerating the replication of EV-A71 in human oral epithelial cells (Fu et al., 2015). Moreover, our previous research confirmed that CV-A16 infection significantly downregulated the expression of miR-1303 in human umbilical vein endothelial cells (HUVECs), which further disrupted junctional complexes by targeting matrix metalloproteinase (MMP9), resulting in increased blood–brain barrier (BBB) permeability and allowing CV-A16 to successfully cross the BBB and enter the central nervous system (CNS), eventually leading to pathological changes in the CNS (Song et al., 2018b). Additionally, we further analyzed the differences in miRNA expression patterns of EV-A71- and CV-A16-infected cell lines, including peripheral blood mononuclear cells (PBMCs) (Song et al., 2018c) of rhesus monkeys, bronchial epithelial cells (16HBE) (Hu et al., 2017; Song et al., 2018a), and HUVECs (Song et al., 2017; Song et al., 2019), which clarified the regulatory roles of miRNAs in the occurrence and development of EV-A71 and CV-A16 infections. Thus, these studies implied that cell-encoded miRNAs directly affect the pathogenesis of enteroviruses, and investigations at the miRNA level could contribute to further understanding of the mechanisms of the interaction between viruses and host cells and provide scientific information for the discovery of novel antiviral agents and strategies. However, there is no report elucidating the detailed role of miRNAs during CV-A10 infection in HUVECs. Furthermore, as mentioned above, CNS complications are among the leading causes of death from CV-A10 infection (Bian et al., 2019). In addition, previous studies have also clearly shown that CNS damage is caused by either the replication of enterovirus itself in the CNS or the immune response activated by enterovirus in the CNS (Gonzalez et al., 2019). HUVECs, as the main component of the BBB, are often used to build models of the BBB *in vitro* for neuropathogenetic mechanistic research on some neurotropic viruses (Untucht et al., 2011), such as Japanese encephalitis virus (JEV) (Ashraf et al., 2021) and West Nile virus (WNV) (Vittor et al., 2020). Therefore, in this work, we infected HUVECs with CV-A10, identified the changes in miRNAs induced by CV-A10 infection through high-throughput sequencing technology, and analyzed the biological functions of these differentially altered miRNAs. This study could help us determine the key miRNAs that cause the neuropathogenesis of CV-A10 infection, which might provide a new target molecule for the diagnosis and treatment of CV-A10 infection.

## Materials and methods

### Cell culture and virus infection

HUVECs were acquired from the American Type Culture Collection (ATCC) and grown in Roswell Park Memorial Institute-1640 (RPMI-1640; Corning, New York, NY, USA) with heat-inactivated 10% foetal bovine serum (FBS; Gibco^®^, Grand Island, NY, USA) plus 100 μg/ml of penicillin and streptomycin at 37°C in a humid incubator with 5% carbon dioxide (CO_2_).

For virus infection, a single-cell suspension at a density of 5 × 10^5^ cells/ml was added to six-well plates. When the cells reached 80% confluence, the cells were incubated with CV-A10 (subgenotype C, GenBank: MN557275) at a multiplicity of infection (MOI) of 1 at 37°C for 2-h absorption. Subsequently, the infected cells were cultured in RPMI-1640 containing 2% FBS and antibiotics at 37°C in an atmosphere of 5% CO_2_ and collected with a cell scraper at 0, 24, and 72 h. The control group in this study was set as cells infected with CV-A10 at 0 hpi.

### RNA extraction, quality control, small RNA library construction, and sequencing

Three replicates from each group were mixed for total RNA extraction according to the instructions of a TRIzol™ reagent (Invitrogen, Carlsbad, CA, USA). Then, the concentration of total RNA was determined using a NanoDrop 2000 spectrophotometer. Moreover, the RNA purity and RNA integrity number (RIN) were evaluated with an Agilent 2100 Bioanalyzer. In addition, only the extracted RNAs with An RIN score≥7.0 and rRNA 28S/18S≥1.6 would be used for subsequent experiments.

Total RNA was purified by polyacrylamide gel electrophoresis (PAGE) to enrich 15~35 nt of molecules. Then, proprietary adapters were ligated to the 5′ and 3′ terminals of the RNA, and the samples were used as templates for cDNA synthesis. The cDNA was amplified using the appropriate number of PCR cycles to produce sequencing libraries, which were subsequently subjected to the proprietary Solexa sequencing-by-synthesis method using the Illumina Genome Analyser (San Diego, CA, USA). Sequencing was carried out at ANOROAD Genome, Inc. (Beijing, China). Eventually, the sequencing data were submitted to the Gene Expression Omnibus (GEO) database (www.ncbi.nlm.nih.gov/geo/) under the accession number GSE236620.

### Bioinformatics identification of sequencing data

#### Analysis of sequencing data

After sequencing, the clean reads were screened from the raw reads by eliminating low-quality and contaminant reads, as follows: 1) low-quality reads (Q30 < 90%), 2) reads without a 3′-primer, 3) reads with 5′-primer contaminants, 4) reads without the insert tag, 5) reads with poly A or T, and 6) reads shorter than 18 nt. Then, sequences of 18~35 nt in length obtained from clean reads of each sample were matched to the reference genome *via* Bowtie V1.1.2. Mapped reads were further mapped to the Rfam database (http://rfam.xfam.org, version 11.0) and RepeatMasker, and the protein-coding genes, repeat sequences, ribosomal RNAs (rRNAs), transfer RNAs (tRNAs), small nuclear RNAs (snRNAs), and small nucleolar RNAs (snoRNAs) were discovered. Then, the remaining small RNA reads were further aligned to the miRNA precursors of the reference species in the miRBase 21.0 database (http://www.mirbase.org/) to identify known miRNAs and aligned to the miReap program (http://sourceforge.net/projects/mireap/) to predict potential novel miRNAs.

#### Expression analysis of miRNAs

For the determination of the influence of CV-A10 infection, miRNA expression levels of CV-A10-infected HUVECs and the controls were calculated and normalized to transcripts per million (TPM) on the basis of the normalization formula TPM = (actual miRNA count/total count of mapped reads) × 10^6^. A differential expression analysis of the two groups was conducted *via* the DEGseq R package. A *p*-value <0.05 and a fold change >2 or <0.5 were set as the thresholds for significantly differential expression. Venn analysis was performed to visualize the number of specific and common differentially expressed miRNAs in different groups. Furthermore, to identify miRNA expression patterns, an unsupervised hierarchical complete linkage cluster analysis and a trend analysis were further carried out.

#### Prediction of potential target mRNAs of miRNAs

The prediction of the targets of differentially expressed miRNAs was conducted with the miRanda (http://www.microrna.org/microrna/home.do), PITA (http://genie.weizmann.ac.il/pubs/mir07/mir07_dyn_data.html), and TargetScan (http://www.targetscan.org/vert_60/) algorithms. These online databases are based on each mathematical algorithm and scoring pattern: 1) context score percentile <50 calculated by TargetScan algorithms, 2) a score of △△G ≥ −10 calculated by PITA algorithms, and 3) max energy >−10 calculated by miRanda algorithms. For high specificity in targeted gene prediction, a gene was viewed as a target gene of differentially expressed miRNAs when the gene was predicted to be a target gene of the miRNA in at least two databases.

#### Functional analysis of predicted miRNA targets

To systemically describe the properties and functions of target genes, functional annotation of predicted miRNA targets was performed by Gene Ontology (GO) and Kyoto Encyclopedia of Genes and Genomes (KEGG) analysis with the Database for Annotation, Visualization and Integrated Discovery (DAVID, https://david.ncifcrf.gov/) online database. GO functional enrichment analysis was used to annotate the target genes regulated by miRNAs from the three aspects of biological process (BP), molecular function (MF), and cellular component (CC) to understand the biological functions, pathways, or cell localization to which these target genes can be enriched. However, KEGG pathway enrichment analysis was used to identify candidate target genes involved in the most important biochemical metabolic and signal transduction pathways. Those with *p*-values ≤0.05 through Bonferroni’s correction were defined as significantly enriched GO and KEGG terms.

#### Regulatory network construction

To identify the key miRNAs probably associated with the pathogenesis of CV-A10, we focused on the overlapping target genes that simultaneously appeared in both GO-BP and Pathway. Then, we used these genes with GeneMANIA online tools to build a gene network according to the potential interrelationships of these genes, including coexpression, colocalization, physical interactions, shared protein domains, pathways, and genetic interactions. Moreover, based on these targeted genes, we, in turn, identified their corresponding miRNAs and constructed the miRNA–mRNA regulatory network through the network visualization and analysis tool Cytoscape software.

### Quantitative reverse transcription polymerase chain reaction verification

In the validation stage, four key miRNAs (namely, hsa-miR-628-5p, hsa-miR-497-5p, hsa-miR-374b-3p, and hsa-miR-32-3p) and their target genes (namely, IL1R1, TLR8, VLDLR, and TENM3) were randomly selected and analyzed using quantitative reverse transcription–polymerase chain reaction (RT–qPCR) (**Table S1**) following the protocols of the PrimeScript RT reagent kit (TaKaRa, Maebashi, Japan) and SYBR Premix Ex Taq kit (TaKaRa, Japan). The expression levels of miRNAs and their target genes were normalized to those of the internal controls U6 and β-actin, respectively. The primers used in this study are listed in **Table S2**. The comparative Ct method (2^−ΔΔCT^) was used to calculate the fold change in gene expression, and the expression data were log2 transformed before analysis. All RT–qPCR experiments were performed in triplicate.

### Cell transfection

The siRNAs for miR-143-3p (si-miR-143-3p) or GFP alone (si-Control) were designed, synthesized, and purchased from Sangon Biotech, Shanghai, China. For transfection of HUVECs with si-miR-143-3p or si-Control, HUVECs were seeded in six-well plates for the first day, and then, the siRNAs were applied as per the manufacturer’s recommended operating manual. Meanwhile, the transfection efficiency was examined by RT–qPCR as above described.

### Western blotting evaluation

Total protein extracts were obtained in radioimmunoprecipitation assay (RIPA) lysis buffer (Beyotime, Shanghai, China) on ice, and Western blotting (WB) assays were performed as described in our previous study (Hu et al., 2018). The antibodies used in this study were as follows: claudin-5 (1:1,000 dilution; Abcam, Waltham, MA, USA), occludin (1:1,000 dilution; Abcam, USA), ZO-1 (1:1,000 dilution; Abcam, USA), VE-cadherin (1:1,000 dilution; Abcam, USA), GAPDH (1:5,000 dilution; Bioworld, Nanjing, China), and horseradish peroxidase-conjugated goat anti-mouse/rabbit IgG secondary antibody (1:2,000 dilution; CST, Danvers, MA, USA). Immunoreactive bands were finally acquired using a chemiluminescence kit in a dark room.

### Immunofluorescence staining

The immunofluorescence (IF) staining technique was used to detect the location of tight junction proteins, and IF staining was carried out as we described previously (Hu et al., 2018). Fluorescence images were eventually captured *via* a confocal microscope (Leica, Wetzlar, Germany).

### Statistics

For sequencing data, raw data from the libraries built by each group were normalized to TPM. For RT–qPCR, the data are expressed as the mean ± standard error of the mean (SEM). Statistical analysis was performed using SPSS 18.0 (SPSS, Inc., USA) and GraphPad Prism 5 (GraphPad Software, USA). A *p*-value of less than 0.05 was considered to indicate a statistically significant difference.

## Results

### Sequencing results of small RNA libraries

**Figure 1:**
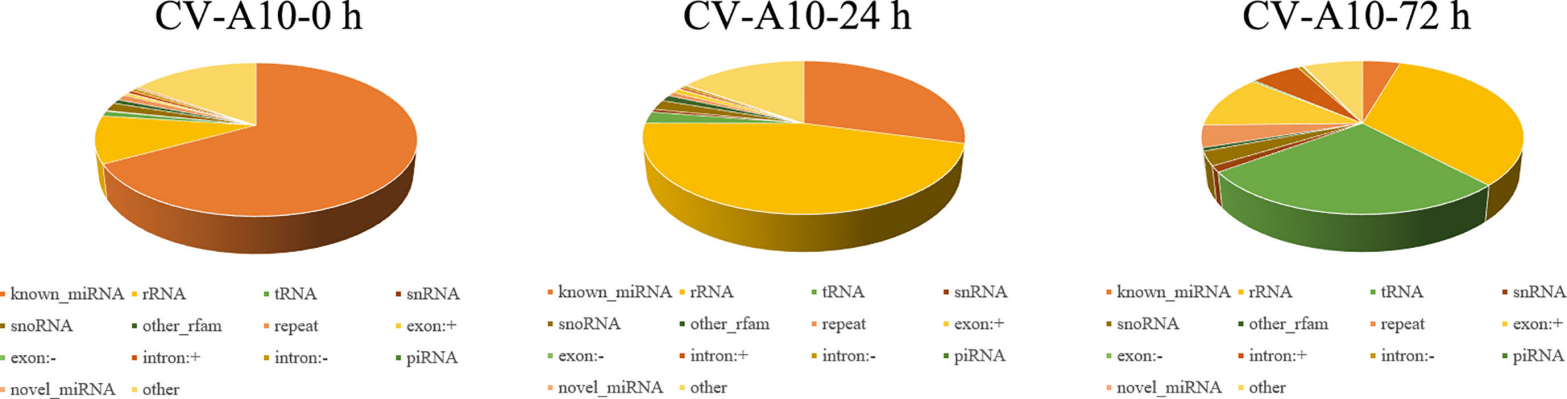
Distribution of the genome-mapped sequence reads in small RNA libraries.

Three small RNA libraries from HUVEC samples consisting of one control and two infected samples were generated. As shown in **Table 1**, 83,849,626, 84,747,872, and 76,480,202 raw reads for the control, CV-A10-24 h, and CV-A10-72 h groups, respectively, were obtained. After stringent data filtering, the remaining clean reads were obtained from the three groups, which contained 7,160,514, 6,431,243, and 12,170,973 clean reads. Moreover, the Q30 values of all groups were greater than 90%, which indicated good sequencing quality of all samples. Subsequently, the perfect match reads and 1 nt-mismatch reads were further identified, and the match rates of all groups were over 80%. Finally, the clean reads were annotated and classified as known miRNA, rRNAs, tRNAs, snRNA, snoRNAs, other_rfam, repeat, exon-sense, exon-antisense, intron-sense, intron-antisense, piRNA, novel miRNAs, and other (**Figure 1**). There were significant differences in the proportion of these classifications in different groups, suggesting that CV-A10 infection might be one of the triggers for obvious composition changes in small RNAs. In addition, detailed numbers of known miRNAs and novel miRNAs are shown in **Table 1**.

**Table 1:** Basic characteristics of small RNA sequences in the three libraries.

Groups	Control	CV-A10-24 h	CV-A10-72 h
Raw reads	83,849,626	84,747,872	76,480,202
Clean reads	7,160,514	6,431,243	12,170,973
Q30 value (%)	92.5	90.71	92.18
Perfect match reads	4,713,703	4,347,773	9,416,483
1 nt-mismatch reads	1,253,442	974,512	1,215,564
Match rate (%)	83.33	82.76	87.36
Known miRNA number	4,000,945	1,552,651	480,322
Novel miRNA number	28,432	10,866	10,198

CV-A10, coxsackievirus A10; miRNA, microRNA.

### CV-A10 infection alters the miRNA expression patterns in HUVECs

**Figure 2:**
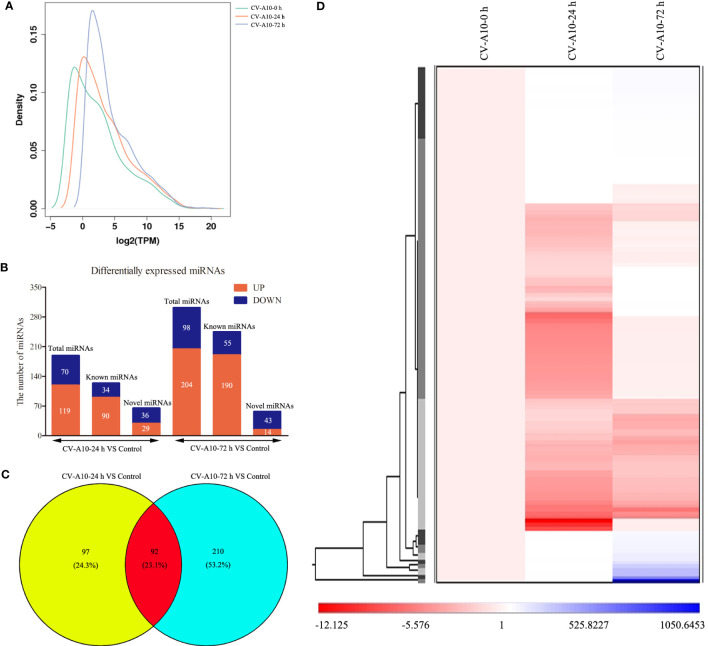
Global analysis of miRNAs. **(A)** Distribution of miRNA expression values in the control, CV-A10-24 h, and CV-A10-72 h groups. **(B)** The number of miRNAs with significantly altered expression is shown. **(C)** A Venn diagram was generated to display the common and specific differentially expressed miRNAs induced by CV-A10 at different infection times. **(D)** Hierarchical cluster of differentially expressed miRNAs during CV-A10 infection at 24 and 72 hpi relative to control samples. MiRNAs, microRNAs; CV-A10, coxsackievirus A10.

As displayed in **Figure 2A**, the overall distribution of miRNA expression values in the three different groups did not overlap, indicating that CV-A10 caused changes in miRNAs. Then, we began to analyze the global cellular miRNA expression patterns of CV-A10 infection. We found that 189 miRNAs were differentially expressed in HUVECs infected with CV-A10 at 24 hpi compared with the controls: 90 upregulated and 34 downregulated known miRNAs, as well as 29 upregulated and 36 downregulated novel miRNAs (**Figure 2B**). Moreover, 302 miRNAs were differentially expressed in the sample infected with CV-A10 at 72 hpi: 190 upregulated and 55 downregulated known miRNAs, as well as 14 upregulated and 43 downregulated novel miRNAs (**Figure 2B**). These above-described miRNAs were also represented using a Venn diagram to reveal the common and distinct differentially expressed miRNAs in different groups. There were 92 overlapping miRNAs across all time points during CV-A10 infection (**Figure 2C**). Thereafter, these common miRNAs were subjected to unsupervised hierarchical clustering analysis with log fold-change values to visually illustrate the expression patterns of the miRNAs during CV-A10 infection over time. The heatmap clearly revealed that miRNA expression levels were significantly different among CV-A10 infection sample groups, showing obvious distinctiveness and clustering (**Figure 2D**). Thus, these findings suggested that differences in the number and expression pattern of miRNAs existed in HUVECs infected with CV-A10 at different times.

### Trend analysis of the common differentially expressed miRNAs

**Figure 3:**
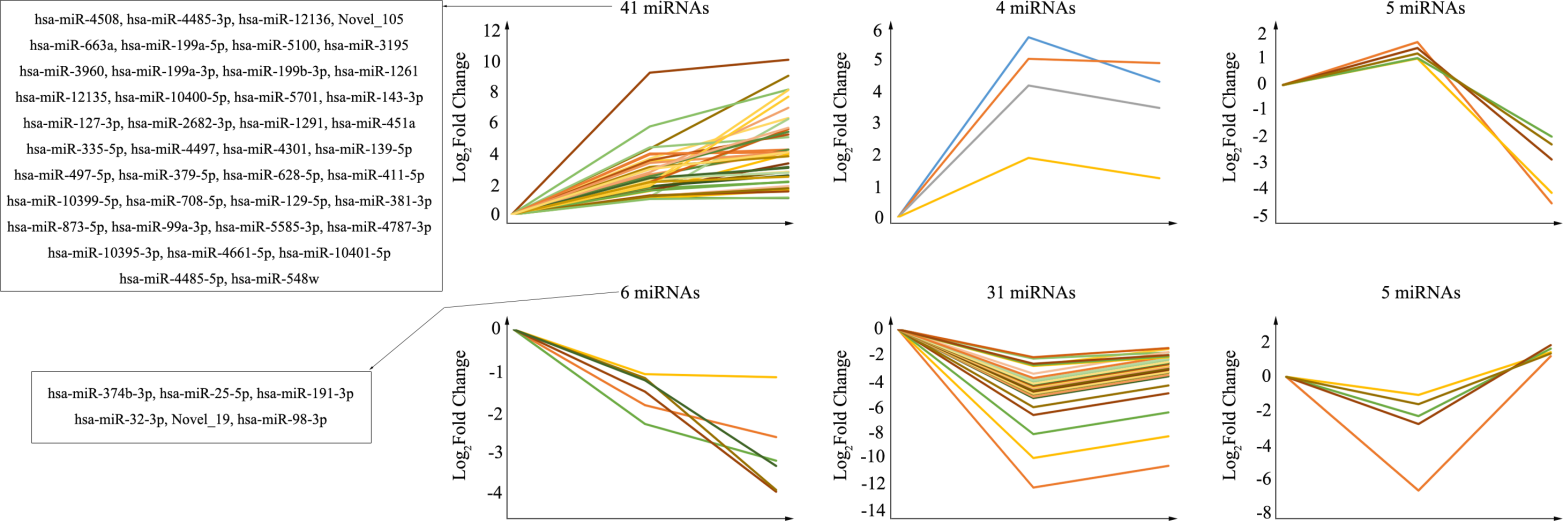
Trend analysis of differentially expressed miRNAs following CV-A10 infection at different time points post-infection. MiRNAs, microRNAs; CV-A10, coxsackievirus A10.

The purpose of trend analysis is to give us a clearer picture of the dynamics of the 92 overlapping miRNAs. These miRNAs showed six distinctly changed trends (**Figure 3**). 1) MiRNAs continued to increase over the duration of infection (41 miRNAs). 2) MiRNAs increased at infection time, but the degree of increase was higher at 24 hpi than at 72 hpi (four miRNAs). 3) MiRNAs were upregulated at 24 hpi but downregulated at 72 hpi (five miRNAs). 4) MiRNAs continued to decrease over the duration of infection (six miRNAs). 5) MiRNAs increased at infection time, but the degree of decrease was higher at 24 hpi than at 72 hpi (31 miRNAs). 6) MiRNAs were downregulated at 24 hpi but upregulated at 72 hpi (five miRNAs). Nevertheless, the miRNAs we focused on were those that were consistently upregulated or downregulated, as those with this same persistent trend over time may be important miRNAs involved in pathological progression following CV-A10 infection. Furthermore, the predicted stem-loop structures of novel miRNAs with a trend of continuous upregulation and downregulation are displayed in **Figure S1**.

### Functional analysis of dysregulated miRNAs

**Figure 4:**
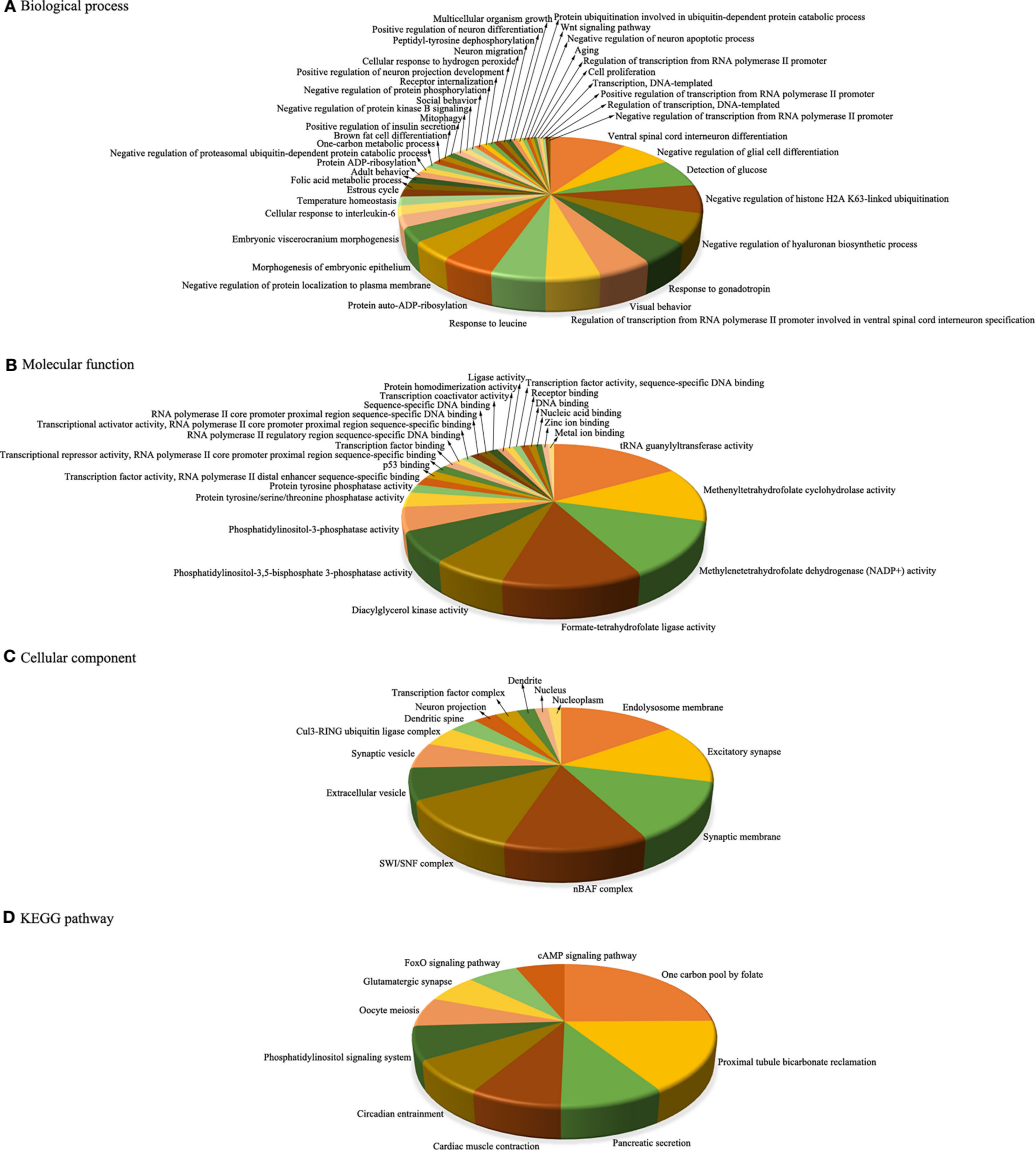
Integrative functional analysis of target genes of persistently upregulated miRNAs. **(A)** Significantly enriched terms based on BP. **(B)** Significantly enriched terms based on MF. **(C)** Significantly enriched terms based on CC. **(D)** Significantly enriched KEGG pathways. MiRNAs, microRNAs; BP, biological process; MF, molecular function; CC, cellular component; KEGG, Kyoto Encyclopedia of Genes and Genomes.

**Figure 5:**
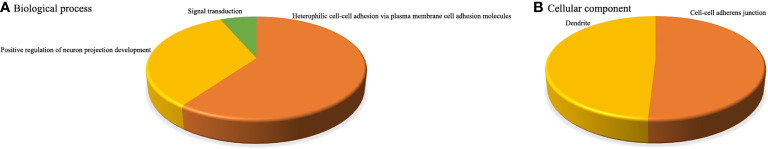
Integrative functional analysis of target genes of persistently downregulated miRNAs. **(A)** Significantly enriched terms based on BP. **(B)** Significantly enriched terms based on CC.

To better understand the potential functions of these key miRNAs, we used the web-based software programs miRanda, PITA, and TargetScan to predict their putative targets. Then, the target genes regulated by aberrantly expressed miRNAs were further subjected to functional enrichment analysis. In the upregulated miRNAs, the corresponding target genes were identified to be enriched in 44 BPs, 26 MFs, 14 CCs, and 10 pathways (**Figure 4**), while in the downregulated miRNAs, the corresponding target genes were identified to be enriched in only three BPs and two CCs, and there were no MFs or pathway enrichment (**Figure 5**). In these enriched GOs and pathways, some basic biological processes (such as cell proliferation, signal transduction, and cAMP signalling pathway), some immune-related mechanisms (such as cellular response to interleukin-6), and some nervous system-related regulations (such as ventral spinal cord interneuron differentiation, negative regulation of glial cell differentiation, neuron migration, and positive regulation of neuron projection development) were clearly visible. Collectively, these results revealed that dysregulated miRNAs might regulate their corresponding target genes involved in basic biological processes, immune-related mechanisms, and nervous system-related regulation throughout infection, all of which were probably related to the occurrence and development of HFMD caused by CV-A10.

### Integrated network analysis

**Figure 6:**
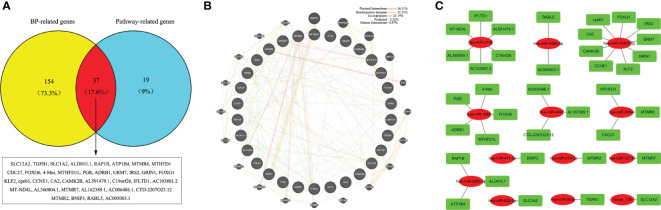
Conjoint analysis of the integrated network in CV-A10 infection. **(A)** Venn diagram of the target genes obtained from the analysis of BP-related genes and pathway-related genes in upregulated miRNAs. **(B)** Coexpression network visualization of target genes that were found to exist in both BP and Pathway. **(C)** The regulatory network based on the upregulated miRNAs and their mRNA targets. CV-A10, coxsackievirus A10; BP, biological process; miRNAs, microRNAs.

**Figure 7:**
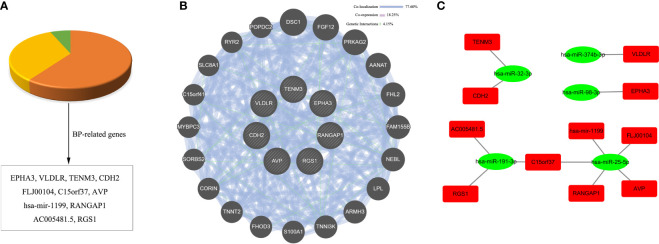
Conjoint analysis of the integrated network in CV-A10 infection. **(A)** Pie chart of the target genes obtained from the analysis of BP-related genes in downregulated miRNAs. **(B)** Coexpression network visualization of target genes that were found to exist in BP. **(C)** The regulatory network based on the upregulated miRNAs and their mRNA targets. CV-A10, coxsackievirus A10; BP, biological process; miRNAs, microRNAs.

To further narrow down the key targets, we identified intersections of related genes in GO-BP and pathway analyses. In doing so, we detected 37 target genes regulated by upregulated miRNAs (**Figure 6A**). Nevertheless, we found no enrichment pathway in downregulated miRNA-mediated targets, so we only focused on GO-BP-related genes (approximately 11 target genes, **Figure 7A**). Then, we used a total of 48 key target genes for coexpression gene network construction. In the coexpression gene networks, there were five functional relations generated by 37 target genes from upregulated miRNAs, namely, physical interaction (36.11%), shared protein domains (32.31%), coexpression (21.19%), predicted (5.52%), and genetic interactions (4.87%) (**Figure 6B**), but there were only three functional relations generated by 11 target genes from downregulated miRNAs, namely, colocalization (77.60%), coexpression (18.25%), and genetic interactions (4.15%) (**Figure 7B**). Afterwards, we searched for miRNAs regulating these target genes again from the data analyzed above and discovered the corresponding 13 upregulated miRNAs and five downregulated miRNAs. Eventually, these miRNAs and their putative target genes further established networks to manifest the complex regulatory role of miRNAs (**Figures 6C**, **7C**).

### Verification of differentially expressed miRNAs and relevant target genes

**Figure 8:**
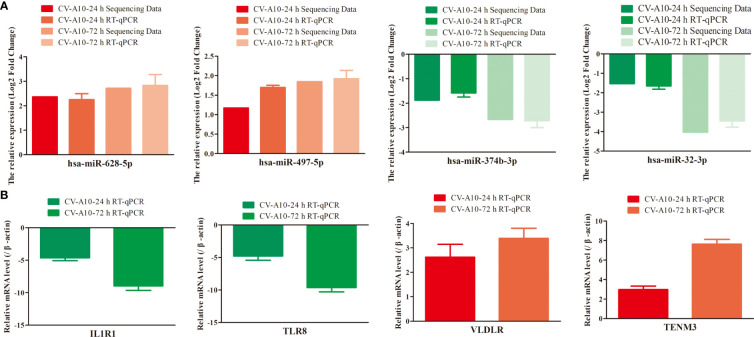
Expression levels of four randomly selected miRNAs **(A)** and their mRNA targets **(B)** were verified by RT–qPCR using independent samples. MiRNAs, microRNAs; RT–qPCR, quantitative reverse transcription–polymerase chain reaction.

To confirm the reliability of our sequencing data, we selected four significantly differentially expressed miRNAs—hsa-miR-628-5p, hsa-miR-497-5p, hsa-miR-374b-3p, and hsa-miR-32-3p—for RT–qPCR analysis (**Table S1**). The results showed a general consistency between the RT–qPCR and high-throughput sequencing results (**Figure 8A**). Furthermore, the expression levels of mRNAs displayed an opposite trend with the miRNAs (**Figure 8B**), which was exactly in line with the theory that miRNAs can exert a negative regulatory effect on their target genes.

### Effects of miR-143-3p on BBB integrity

**Figure 9:**
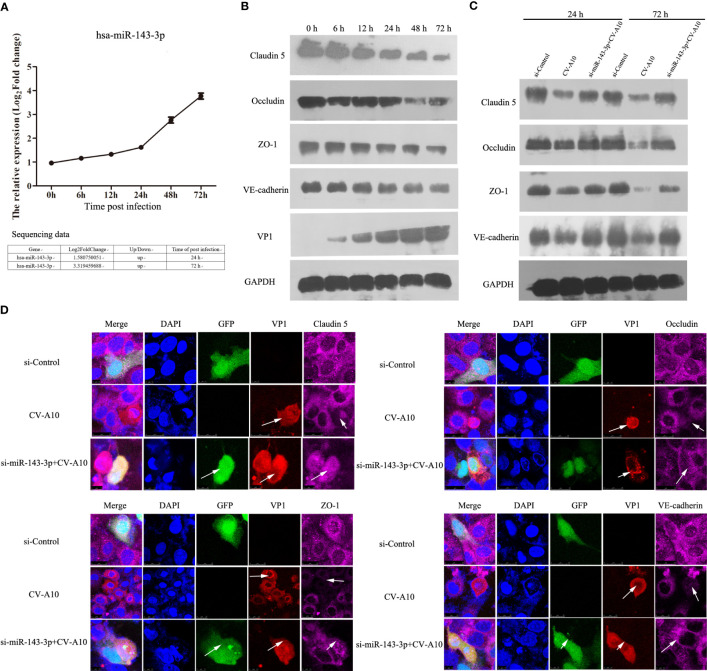
The influence of miR-143-3p on junction proteins. **(A)** Hsa_miR_143-3p expression was measured by qRT–PCR. **(B)** The expression levels of junctional proteins were examined by WB in CV-A10-infected HUVECs. **(C)** The expression of junctional proteins in HUVECs treated with si-Control vector, CV-A10 infection, and si-miR-143-3p vector plus CV-A10 infection was identified with WB at 24 and 72 hpi. **(D)** Confocal imaging showed the junctional protein localization of claudin-5, occludin, ZO-1, and VE-cadherin at 72 hpi. RT–qPCR, quantitative reverse transcription–polymerase chain reaction; WB, Western blotting; CV-A10, coxsackievirus A10; HUVECs, human umbilical vein endothelial cells.

Among these dysregulated miRNAs, miR-143-3p attracted our attention because miR-143 has been verified to be involved in methamphetamine-induced BBB disruption by targeting p53 unregulated modulator of apoptosis (PUMA), which leads to a decrease in tight junction molecules, such as claudin-5, occludin, and ZO-1 (Wang et al., 2021). We next monitored the expression of miR-143-3p and showed that miR-143-3p was significantly increased after CV-A10 infection (**Figure 9A**). Moreover, junction molecules, namely, claudin-5, occludin, ZO-1, and VE-cadherin, which are essential proteins for maintaining the permeability of the BBB (Abbott et al., 2010), were dramatically decreased in response to CV-A10 infection (**Figure 9B**). Thereafter, to further explore the role of miR-143-3p in BBB integrity, we transfected si-miR-143-3p into cells. The RT–qPCR result showed that the expression of miR-143-3p was markedly declined after transfecting with the si-miR-143-3p plasmid (**Figure S2**). Then, it was further found that silencing miR-143-3p improved the expression of claudin-5, occludin, ZO-1, and VE-cadherin at 48 and 72 hpi (**Figure 9C**). Moreover, double IF staining of junction molecules and VP1 proteins indicated that the location of junction molecules was remarkably destroyed by CV-A10 infection, but the destruction of junction molecules was ameliorated in the si-miR-143-3p+CV-A10 group (**Figure 9D**). Taking these results together, we proposed that miR-143-3p might exert a negative regulatory role in preserving BBB integrity.

## Discussion

CV-A10, as one of the major pathogens causing HFMD, has been found to be closely associated with severe HFMD outbreaks in mainland China in recent years (Bian et al., 2019), but there are no specific treatments for CV-A10-associated HFMD (Aswathyraj et al., 2016); therefore, prevention, diagnosis, control, treatment, and rehabilitation of CV-A10-associated HFMD are challenging. However, accumulating evidence indicates that virus infection can mediate changes in the expression of cellular miRNAs, which can not only lead to downstream changes in the host transcriptome that can be advantageous to the virus and can also lead to increases in antiviral effector activities, resulting in decreased viral replication (Duan et al., 2020). In addition, host miRNAs can bind to RNA viral genomes, regulating their translation and replication and altering viral pathogenesis (Trobaugh and Klimstra, 2017). Consequently, it is indisputable that miRNAs play critical roles in the regulation of viral infections and antiviral responses (Sedger, 2013). In recent years, high-throughput sequencing has become a powerful strategy for identifying novel miRNAs and studying the expression profiles of miRNAs in different samples (Pritchard et al., 2012; Backes et al., 2016). In this work, we successfully identified differentially expressed miRNAs in HUVECs against CV-A10 infection using high-throughput sequencing. Furthermore, 189 and 302 differentially expressed miRNAs were found at 24 and 72 h after CV-A10 infection, respectively. Thus, these changes in miRNA expression patterns suggested that miRNAs may have a profound effect on the host response after CV-A10 infection. Subsequently, for validation of the sequencing data, RT–qPCR was carried out on four randomly selected miRNAs. The changes in miRNA expression assayed using sequencing and RT–qPCR were basically consistent, although variation in the magnitude of expression was observed, possibly because expression by the two methods is influenced by data quality parameters and the relative amount of change. Moreover, according to the negative regulatory relationship between miRNAs and their targets, we conducted RT–qPCR verification on the target molecules corresponding to these four miRNAs, and the results showed that the expression trend of these target molecules and the upstream miRNAs was the opposite.

To better screen out miRNAs involved in the whole process of CV-A10 infection, we focused on overlapping miRNAs at 24 and 72 h after CV-A10 infection. We then performed unsupervised hierarchical clustering and trend analysis of these overlapping miRNAs. The results showed that 41 miRNAs were continuously upregulated and six miRNAs were continuously downregulated during CV-A10 infection; these molecules were considered key miRNAs for subsequent exploration. Subsequently, the target genes of these persistently changed miRNAs were further predicted, and the functions of the putative target genes were also analyzed. Bioinformatics analysis indicated that the predicted target genes were mainly linked to basic biological processes, immune-related mechanisms, and nervous system-associated regulation, and these functional changes were thought to be directly related to the pathogenesis of CV-A10 infection. Next, we discuss the possible importance of some enriched GOs and pathways after CV-A10 infection. GO annotation for target genes offers a better understanding of the target genes at the biological, molecular, and cellular levels (Ding et al., 2018), whereas KEGG pathway analysis for target genes provides potential signalling pathway transduction mechanisms of the target genes (Kanehisa et al., 2017). First, cell proliferation is critical for understanding the mechanisms of action of certain genes, proteins, and pathways involved in cell survival or death after exposure to virus infection (Chen et al., 2020). For example, inhibition of cell proliferation is evident in cells infected with the chikungunya virus (CHIKV) (Sharma et al., 2015). Human cytomegalovirus (HCMV) infection enhances cell proliferation in colorectal cancer-derived stem cell-like cells (Teo et al., 2017). In addition, mounting studies have reported that miRNAs play an important role in the regulation of cell proliferation in many viral diseases, especially virus-mediated cancers, such as hepatitis B virus (HBV) (Oura et al., 2020). Thus, the enriched “cell proliferation” mediated by differentially expressed miRNAs in the present work might exert an essential physiological function in CV-A10 infection. Second, interleukin 6 (IL-6), which has emerged as a master regulator of inflammation, is involved in innate and adaptive immune responses to defend against pathogens (Choy et al., 2020). For instance, significantly higher levels of IL-6 were detected in children with H1N1 virus infection, and the upregulation of IL-6 likely plays a proinflammatory role in H1N1 virus infection, which might be the crucial factor for airway inflammation and bronchial hyperreactivity in these children (Chiaretti et al., 2013; Zhao et al., 2021). IL-6 was also found to be a key member in triggering cytokine storms during coronavirus disease 2019 (COVID-19) infection (Coomes and Haghbayan, 2020; Liu B. et al., 2020). Additionally, IL-6 has been reported to be associated with severe EV-A71 infection by numerous research teams (Luo et al., 2019; Zhang et al., 2020). Moreover, researchers have further proven that IL-6-mediated signalling, as a vital component of innate immunity and inflammation, could be negatively regulated by miRNAs at the post-transcriptional level during viral infection, such as rabies virus (RABV) (Tu et al., 2019). Therefore, the enriched “cellular response to interleukin-6” regulated by differentially expressed miRNAs in the present study might be primarily related to the immune or inflammatory response induced by CV-A10 infection. Third, extensive literature has reported that CV-A10 outbreaks are even more prevalent in Asia and that CV-A10 infection usually causes more severe forms of HFMD, especially triggering CNS complications (Bian et al., 2019). However, the key pathogenesis of central nervous system complications triggered by CV-A10 infection is still unknown. In this study, it was observed that there were a large number of neurosystem-related regulations in the enriched GOs and pathways, such as “ventral spinal cord interneuron differentiation”, “negative regulation of glia cell differentiation”, “neuron migration”, “positive regulation of neuron differentiation”, “negative regulation of neuron apoptotic process”, and “positive regulation of neuron projection development”, which implied that these processes might directly participate in the neuropathogenesis of CV-A10 infection. Furthermore, in CC analysis, we found that many nervous system-related cellular components were enriched, namely, “excitatory synapse”, “synaptic membrane”, “synaptic vesicle”, “dendritic spine”, “neuron projection”, and “dendrite”, which further suggested that a significant change existed in the nervous system during CV-A10 infection. Altogether, the above enrichment analysis has ultimately clarified that the pathogenesis of HFMD induced by CV-A10, especially immune dysregulation and neurological impairment, might be closely correlated with abnormally expressed miRNA-mediated regulatory mechanisms.

Afterwards, to further determine the effects of key miRNAs, we identified the target genes involved in both BPs and pathways. We deeply analyzed the interaction networks of these genes and their corresponding miRNAs. First, we established a gene coexpression network according to the extensive functional association data. We found five and three regulatory relationships in target genes of up- and downregulated miRNAs, respectively. Then, we also built miRNA regulatory networks according to the relationships between the dysregulated miRNAs and their targets. Based on the theory that miRNAs can specifically bind to the 3′-non-coding regions of mRNA and then play a negative regulatory role in degrading mRNA or inhibiting translation, it is believed that miRNAs can indirectly suppress their target-related biological processes or pathways by regulating their downstream targets (Cai et al., 2009). Among the miRNA regulatory networks, we arbitrarily selected the miR-663a–TGFB1 axis and the miR-4286–FOXO4 axis for further discussion. The miR-663a–TGFB1 axis was an upregulated miRNA-mediated regulatory axis, while the miR-4286–FOXO4 axis was a downregulated miRNA-mediated regulatory axis. In fact, in previous studies, the targeted regulatory interaction of miR-663a and TGFB1 has been demonstrated (Zhang et al., 2018). Moreover, numerous studies have previously revealed that changes in TGFB1-regulated signalling have pivotal influences on viral infections, such as Epstein–Barr virus (EBV) (Filatova et al., 2019), respiratory syncytial virus (RSV) (Xu et al., 2020), and COVID-19 (Vaz de Paula et al., 2021), thereby suggesting that the upstream regulatory molecule miR-663a of TGB1 may directly affect the infection process of these viruses. Additionally, miR-4286 is believed to have a tumour promoter role in the progression of numerous cancers, including non-small cell lung cancer (An et al., 2019), gastric cancer (Tsai et al., 2020), and prostate cancer (Li et al., 2019), but whether miR-4286 has a vital role in virus infection remains to be investigated. However, its predicted target gene FOXO4 has shown aberrant expression in viral infections, such as HBV (Srisuttee et al., 2011) and EBV (Liu W. et al., 2020), in many investigations. Hence, the miR-4286–FOXO4 axis might be a key regulatory point during virus infection. Overall, the analysis of the above networks highlighted the key role of altered miRNAs in the regulation of the host response, which could further assist us in uncovering the possible mechanisms of miRNAs in the occurrence and development of CV-A10 infection.

Finally, among these key dysregulated miRNAs, we focused on miR-143-3p, mainly because miR-143 has been proven to play an important role in central nervous system diseases. For example, circulating miR-143-3p was reported to be elevated in both derivation and validation cohorts of acute stroke (Tiedt et al., 2017), and miR-143 was also demonstrated to be associated with neurological deficits, infarct areas, and BBB extravasation (Bai et al., 2018). Moreover, methamphetamine mediates the upregulation of miR-143, which further increases the permeability of human brain endothelial cells and concomitantly decreases the expression of tight junction proteins by targeting PUMA, leading to the disruption of BBB integrity and accelerating the neuroinflammation induced by methamphetamine (Bai et al., 2016). In this work, we observed that miR-143-3p was also enhanced; therefore, we hypothesized that miR-143-3p might exert a negative regulator for the maintenance of BBB integrity during CV-A10 infection. The BBB, composed of endothelial cells, pericytes, astrocytic end feet, and basement membranes, prevents neurotoxic plasma components, blood cells, and pathogens from entering the brain (Abbott et al., 2010). Strong evidence has indicated that disruption of the integrity of the structure and function of the BBB is a prerequisite for most neurotropic viruses to invade the CNS, including enteroviruses (Al-Obaidi et al., 2018). Furthermore, miRNAs were found to be enriched in the CNS, and an increasing number of studies have also reported that miRNAs are essential mediators for the regulation of BBB permeability (Wang et al., 2021). Moreover, our previous study verified that miR-1303 could regulate BBB permeability and promote CNS lesions following CV-A16 infection by directly targeting MMP9 (Song et al., 2018b). Hence, in this work, we used HUVECs, which are usually used to build an *in vitro* model of the BBB in neurotropic virus infection (Ogunshola, 2011), to examine the expression of miR-143-3p and junction proteins following CV-A10 infection. MiR-143-3p was gradually increased, but the junction proteins were gradually decreased. Moreover, we found that the locations of junction proteins disappeared after CV-A16 infection, while silencing miR-143-3p markedly improved the expression of junction proteins and notably recovered the locations of junction proteins. Junction complexes between cerebral endothelial cells are essential for maintaining BBB integrity and mainly contain tight junctions (e.g., claudins, occludins, and ZOs) and adherens junctions (e.g., VE-cadherin, α-catenin, and β-catenin) (Abbott et al., 2010). Thus, our results suggested that miR-143-3p might be involved in the destruction of BBB integrity by destroying the junction complexes during CV-A10 infection.

Accordingly, this is the first study to systematically compare the miRNA profile of CV-A10-infected HUVECs to that of uninfected controls. We identified a total of 189 and 302 differentially expressed miRNAs in CV-A10-infected cells at 24 h and 72 h, respectively. Then, target prediction and bioinformatics analysis showed that these differentially expressed miRNAs were likely to be critical regulators of the host immune response and neuropathogenesis during CV-A10 infection. Ultimately, the construction of the network further revealed the regulatory roles of miRNAs in host–CV-A10 interactions. Moreover, *in vitro* results confirmed that miR-143-3p might disrupt BBB permeability during CV-A10 infection by regulating junction proteins. Collectively, these miRNAs might provide new insight for the further elucidation of the pathogenesis of CV-A10 infection, for the screening of auxiliary screening markers of CV-A10 diagnosis, and the development of therapeutic drugs for CV-A10 treatment.

## Data availability statement

The datasets presented in this study can be found in online repositories. The names of the repository/repositories and accession number(s) can be found below: https://www.ncbi.nlm.nih.gov/geo/, GSE236620.

## Author contributions

YH: Conceptualization, Writing-original draft, Funding acquisition. FC: Methodolog, Software, Data curation. SW: Methodology, Software. CL: Methodology. SZ: Visualization, Investigation. RW: Visualization, Investigation. JS: Conceptualization, Writing-review and editing, Funding acquisition. YZ: Conceptualization, Supervision, Writing-review and editing. All authors contributed to the article and approved the submitted version.

## Supplementary material

Supplementary Table 1 Selected miRNAs for RT-qPCR.

Supplementary Table 2 Primer sequences for analysis of miRNAs and their target gene expression using RT-qPCR.

Supplementary Figure 1 Predicted stem-loop structure of novel differentially expressed miRNAs.

Supplementary Figure 2 Validation of miR-143-3p expression by RT-qPCR after transfecting with miR-143-3p knockout plasmid.
