# Supplementary material for: MicroRNA expression profile of human umbilical vein endothelial cells in response to coxsackievirus A10 infection reveals a potential role of miR-143-3p in maintaining the integrity of the blood–brain barrier
Source: Front Cell Infect Microbiol. 2023 Jul 28;13:1217984. doi: 10.3389/fcimb.2023.1217984 (PMC10419304; doi:10.3389/fcimb.2023.1217984)
Supplement: Supplementary Table 1 — Selected miRNAs for RT-qPCR. [file Table_1.docx]

**Table S1.** Selected miRNAs for RT-qPCR.

| **miRNA** | **Regulation direction** | **CV-A10-24 h** | **CV-A10-72 h** | **Target genes** |
| --- | --- | --- | --- | --- |
|  |  | **Log_2_FoldChange** | **Log_2_FoldChange** |  |
| **hsa-miR-628-5p** | Up | 2.363938662 | 2.715131863 | DNAJC15, IL1R1, SLC1A2 |
| **hsa-miR-497-5p** | Up | 1.171293584 | 1.84441488 | A2MP1, ZBTB46, TLR8, ITGA3 |
| **hsa-miR-374b-3p** | Down | -1.877069437 | -2.66337976 | LOC101929268, MIR374B, VLDLR |
| **hsa-miR-32-3p** | Down | -1.532225527 | -4.029029233 | TENM3, B3GNT5, CDH2 |

**Table S2.** Primer sequences for analysis of miRNAs and their target genes expression using RT-qPCR.

| **miRNAs or Target genes** | **Primers** |
| --- | --- |
| **hsa-miR-628-5p** | F：5’-CGCAGATGCTGACATATTTAC-3’  R：5’-GGTCCAGTTTTTTTTTTTTTTTCCT-3’ |
| **hsa-miR-497-5p** | F：5’-GCAGCAGCACACTGT-3’  R：5’-CCAGTTTTTTTTTTTTTTTACAAACCA-3’ |
| **hsa-miR-374b-3p** | F：5’-GCAGCTTAGCAGGTTGTATTATC-3’  R：5’-CAGGTCCAGTTTTTTTTTTTTTTTAATG-3’ |
| **hsa-miR-32-3p** | F：5’-GCAGCAATTTAGTGTGTGTG-3’  R：5’-CAGGTCCAGTTTTTTTTTTTTTTTAAATATC-3’ |
| **has-miR-143-3p** | F：5’- GGGGTGAGATGAAGCACTG-3’  R：5’- CAGTGCGTGTCGTGCAGT-3’ |
| **U6** | F：5’-CAGTGCGTGTCGTGCAGT-3’  R：5’-AACGCTTCACGAATTTGCGT-3’ |
| **IL1R1** | F：5’-AGCAGAAACTACCCGTTGCAG-3’  R：5’-TTCAGCCACATTCATCACGAT-3’ |
| **TLR8** | F：5’-CCTTAATAGGCTGAAGCACA-3’  R：5’-GTTTCCACGTAAGTCAAGCAA-3’ |
| **VLDLR** | F：5’-ATGCCAAAATCCAGGAATCTGC-3’  R：5’-AGATCCATTTGATAGCCACGACT-3’ |
| **TENM3** | F：5’-TGGCCTATACTTTCATATGGGA-3’  R：5’-CAGAGTCAGGTCCAAACACGA-3’ |
| **β-actin** | F：5’-GGGCATGGGTCAGAAGGATT-3’  R：5’-TCGATGGGGTACTTCAGGGT-3’ |
