# Supplementary figures and images for: MicroRNA expression profile of human umbilical vein endothelial cells in response to coxsackievirus A10 infection reveals a potential role of miR-143-3p in maintaining the integrity of the blood–brain barrier
Source: Front Cell Infect Microbiol. 2023 Jul 28;13:1217984. doi: 10.3389/fcimb.2023.1217984 (PMC10419304; doi:10.3389/fcimb.2023.1217984)

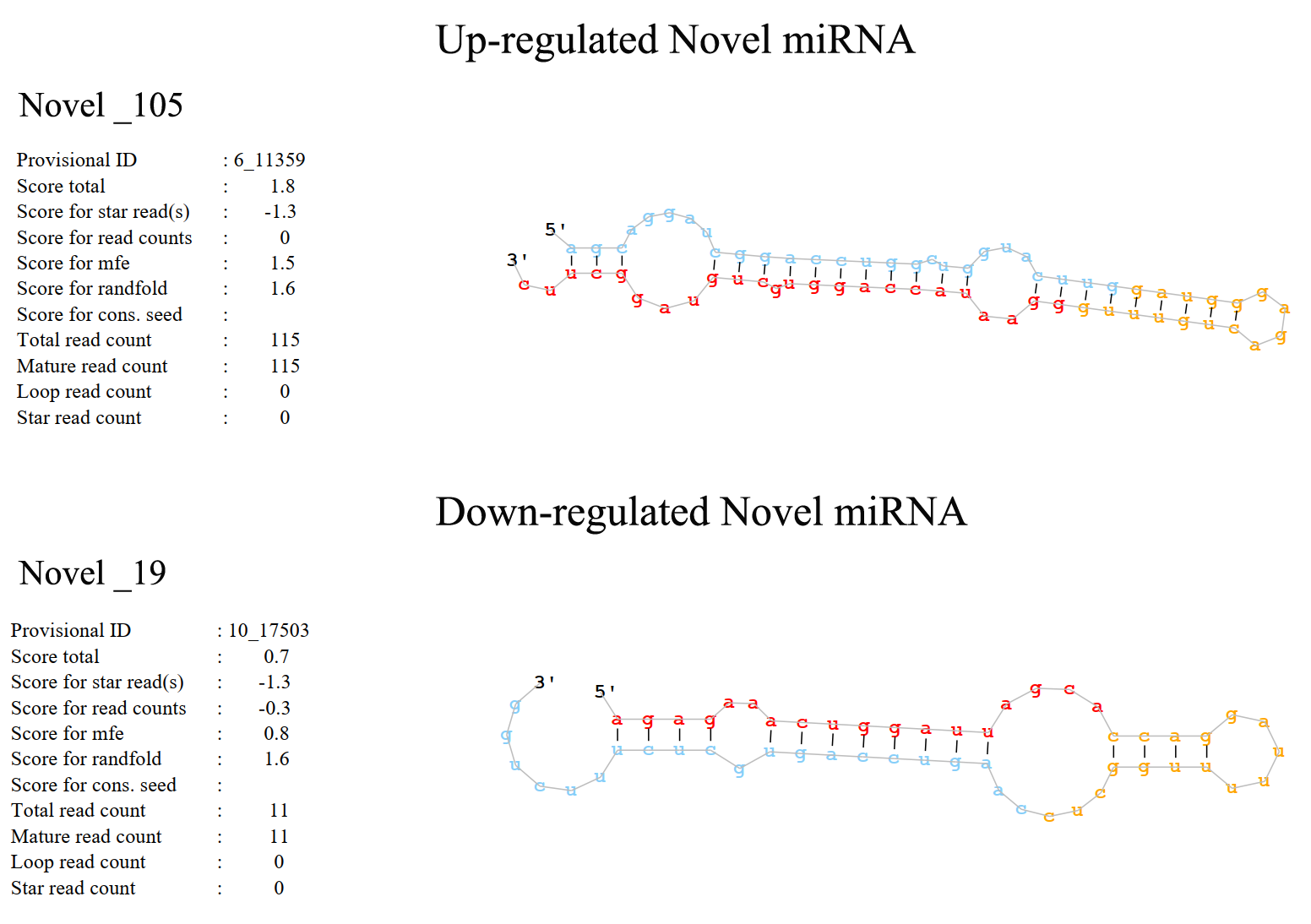

Supplement: Supplementary Figure 1 — Predicted stem-loop structure of novel differentially expressed miRNAs. [file Image_1.tif]

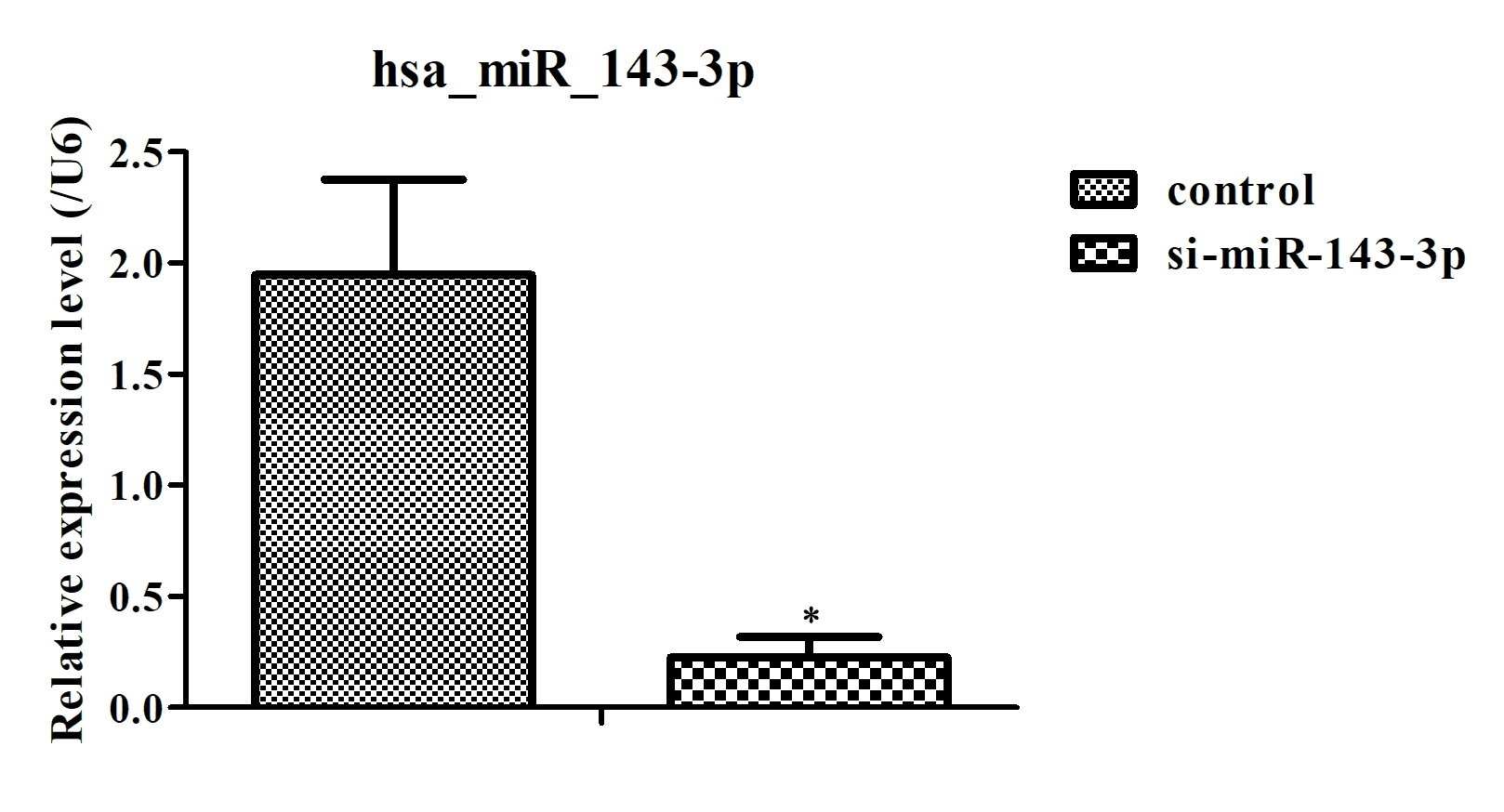

Supplement: Supplementary Figure 2 — Validation of miR-143-3p expression by RT-qPCR after transfecting with miR-143-3p knockout plasmid. [file Image_2.tif]
